# Detection Model of Tea Disease Severity under Low Light Intensity Based on YOLOv8 and EnlightenGAN

**DOI:** 10.3390/plants13101377

**Published:** 2024-05-15

**Authors:** Rong Ye, Guoqi Shao, Ziyi Yang, Yuchen Sun, Quan Gao, Tong Li

**Affiliations:** 1 College of Food Science and Technology, Yunnan Agricultural University, Kunming 650201, China; 15912913557@163.com; 2 The Key Laboratory for Crop Production and Smart Agriculture of Yunnan Province, Kunming 650201, China; 15751769522@163.com (G.S.); yangziyi5199@163.com (Z.Y.); 15687760971@163.com (Y.S.); 3 Big Data College, Yunnan Agricultural University, Kunming 650201, China

**Keywords:** EnlightenGAN, YOLOv8, tea diseases, object detection, deep learning, low light intensity

## Abstract

In response to the challenge of low recognition rates for similar phenotypic symptoms of tea diseases in low-light environments and the difficulty in detecting small lesions, a novel adaptive method for tea disease severity detection is proposed. This method integrates an image enhancement algorithm based on an improved EnlightenGAN network and an enhanced version of YOLO v8. The approach involves first enhancing the EnlightenGAN network through non-paired training on low-light-intensity images of various tea diseases, guiding the generation of high-quality disease images. This step aims to expand the dataset and improve lesion characteristics and texture details in low-light conditions. Subsequently, the YOLO v8 network incorporates ResNet50 as its backbone, integrating channel and spatial attention modules to extract key features from disease feature maps effectively. The introduction of adaptive spatial feature fusion in the Neck part of the YOLOv8 module further enhances detection accuracy, particularly for small disease targets in complex backgrounds. Additionally, the model architecture is optimized by replacing traditional Conv blocks with ODConv blocks and introducing a new ODC2f block to reduce parameters, improve performance, and switch the loss function from CIOU to EIOU for a faster and more accurate recognition of small targets. Experimental results demonstrate that YOLOv8-ASFF achieves a tea disease detection accuracy of 87.47% and a mean average precision (mAP) of 95.26%. These results show a 2.47 percentage point improvement over YOLOv8, and a significant lead of 9.11, 9.55, and 7.08 percentage points over CornerNet, SSD, YOLOv5, and other models, respectively. The ability to swiftly and accurately detect tea diseases can offer robust theoretical support for assessing tea disease severity and managing tea growth. Moreover, its compatibility with edge computing devices and practical application in agriculture further enhance its value.

## 1. Introduction

Tea, a traditional beverage, has garnered significant attention in the market [1]. However, with the increasing demand for tea and the expansion of production, the prevalence of tea diseases has also risen, significantly impacting tea yield and quality. In Yunnan large-leaf tea plants, there are approximately 100 types of tea tree diseases, with more than 30 being relatively common, such as tea anthracnose and tea moire leaf blight, which severely hinder the growth of tea trees, leading to decreased yield and quality. Furthermore, as these diseases progress, the use of pesticides and harmful substances may result in residues in the tea, potentially endangering consumers’ health and safety [2].

In addressing tea diseases, it is essential to implement intelligent, accurate, and efficient disease prevention and control measures. The field of crop disease detection has gained significance with the progress of agricultural technology [3]. While traditional machine learning methods have been extensively researched, they do not offer efficient automatic disease identification. Therefore, it is imperative to steer the advancement of tea garden disease prevention and control towards intelligent solutions to enhance tea production and quality as well as to safeguard the health and safety of consumers [4,5,6,7,8].

As the intelligentization process of modern agriculture progresses, deep learning technology has proven to be highly advantageous in crop disease detection [9]. Deep learning algorithms, in contrast to traditional machine learning methods, exhibit high recognition accuracy and strong robustness and are unaffected by environmental factors, making them particularly well suited for disease detection in large-leaf tea. Researchers [10,11,12,13,14,15,16,17,18] have made significant advancements by refining algorithms, such as integrating SLIC and SVM algorithms, utilizing depthwise separable convolution and ResNet models, and employing conditional convolutional generative adversarial networks (C-DCGAN). These innovations not only enhance the accuracy of tea disease detection but also address the time-consuming nature of manual observation, offering more efficient solutions for agricultural production. However, in complex tea disease detection scenarios, while convolutional neural networks effectively represent local features, they may struggle to capture the global correlation information across distant pixels [19].

Deep learning still encounters several challenges and issues in crop leaf disease detection within complex environments. First, model complexity and high computing resource consumption present significant obstacles. The algorithms used for crop leaf disease detection often involve extensive calculations and parameter requirements, leading to elevated costs that may not always align with the benefits in practical agricultural settings. The substantial demand for computing resources hinders the widespread implementation of these algorithms. Therefore, there is a need to explore more lightweight models and algorithms to reduce costs and improve deployability. Second, the difficulty in feature extraction due to occlusion poses a major challenge. Leaf disease targets are frequently obscured by vegetation and leaves [20,21,22], resulting in an abundance of redundant features. This obscuration diminishes the visibility of crucial features of the target and impairs the feature extraction capabilities of computer vision models. Occlusion complicates leaf disease detection, necessitating a more adaptive and robust algorithm to identify partially occluded leaf disease targets. Lastly, the issue of image noise interference is a significant concern. Images utilized for crop leaf disease detection may contain various noise interferences from soil, weeds, fluctuations in lighting, and multiple types of leaf diseases, making it challenging for computer vision models to accurately classify and locate different leaf diseases. These characteristics often lead to missed detections. Implementing effective noise suppression technology is crucial for enhancing the accuracy of crop leaf disease detection algorithms.

The urgent development of modern smart agriculture necessitates the use of more efficient, lightweight, and robust crop leaf disease detection algorithms. These algorithms must be able to overcome occlusion and adaptive noise in order to provide practical solutions for smart agriculture, ultimately ensuring the quality and yield of crops.

This study addresses the challenges of low disease recognition rates and complex feature extraction in traditional visual detection models by optimizing the structure of deep learning target detection networks. Specifically focusing on the improvement and optimization of models for three major tea diseases in the high temperature and high humidity region of Yunnan tea leaf blight (Exobasidium vexans), tea white spot disease (Exobasidium japonicum), and tea coal disease (Exobasidium camelliae). Effective disease control is crucial for the growth, quality, and safety of tea trees, ultimately impacting the tea-drinking experience. Among them, tea leaf blight, caused by a specific fungus, typically occurs during May–June and September–October. The initial symptoms include small yellow-brown spots on leaf tips and edges, which then expand and turn brown, often in semicircular or irregular shapes. Dark brown lines may appear at the junction between diseased and healthy areas. Severe cases may result in gray and withered leaves. Tea white spot disease, caused by tea leaf point mold, is common in high mountain tea gardens in Yunnan. It mainly affects young leaves and buds, with a fast infection rate. Infected leaves may have a higher breakage rate during processing, resulting in bitter, dark tea soup with a low aroma. Tea sooty disease is more likely to occur in low-temperature, humid environments with serious insect infestations, primarily affecting young leaves. Symptoms include small, black, round or irregular spots that gradually expand, turning into black, sooty spots in severe cases. This soot-like substance can cover the entire leaf, spreading to twigs and stems, giving the plant a dirty, black appearance. Cutting-edge technologies such as deep learning have promising applications in smart agricultural production, particularly in precise disease identification. These technologies can support automatic detection and algorithm development for Yunnan large-leaf tea diseases.

## 2. Materials and Methods

### 2.1. The Image Enhancement Algorithm

Generative adversarial network (GAN) is a deep learning model [23,24,25] utilized for generating new data. GAN comprises a generator and a discriminator. When capturing and storing images of diseased tea leaves, noise can be introduced, impacting the identification of diseased spots. Hence, this study employs EnlightenGAN to enhance images of tea disease samples taken under low-light conditions, minimizing noise, enhancing image quality, and utilizing them as foundational data for further image processing.

### 2.2. Improve the EnlightenGAN Algorithm

By analyzing previous image enhancement algorithms, it has been observed that many of them heavily depend on using pairs of damaged and high-quality images for training. This approach often results in model overfitting and a lack of generalization ability. In order to address this issue, EnlightenGAN is proposed as a method based on unsupervised learning.

**Figure 1:**
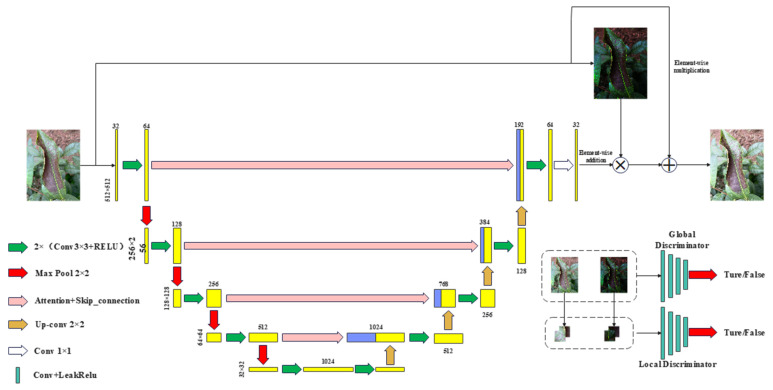
An EnlightenGAN-enhanced model structure based on low-light images.

EnlightenGAN has demonstrated strong performance in overall metrics and enhancing visual effects in low-light scenarios. However, it still faces challenges related to noise amplification in extremely dark areas, insufficient retention of enhanced detail information, and the presence of unknown artifacts when downgrading operations are applied. Additionally, EnlightenGAN struggles to eliminate unknown artifacts and prevent underexposure or overexposure in low-light images with complex backgrounds. The EnlightenGAN network differs from traditional image enhancement methods by incorporating two channels for input: the original root image and the labeled image. The training structure, illustrated in Figure 1, involves a generator that produces raw root images and annotations to reconstruct images and a discriminator that differentiates between input images from the generator. Additionally, the input image resolution can be increased and image brightness enhanced to align with the original image.

In response to the limitations of the previous EnlightenGAN model, enhancements were implemented. One improvement involved the incorporation of the Residual Swin Transformer Layer module, which is capable of capturing long-range feature dependencies in input images using fewer parameters while also reducing noise and artifacts.

**Figure 2:**
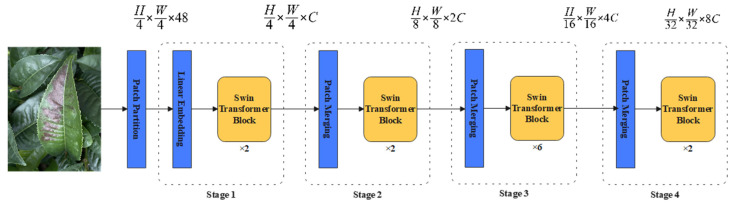
Diagram of Swin Transformer structure.

Transformer introduced a self-attention mechanism to capture global contextual information and improve performance across various vision tasks. Swin Transformer, similar to Transformer, utilizes self-attention to understand relationships between different elements. By employing a hierarchical construction approach, a hierarchical transformer is created, allowing for nodes to have a larger receptive field as the network deepens. Self-attention calculations are carried out in overlapping windows to reduce computational complexity and address the issue of limited global impact. Additionally, the local self-attention mechanism enables the processing of large images. By utilizing window schemes with rule division and shift division, long-distance feature dependencies can be effectively modeled while reducing computational load and enhancing modeling capabilities. The Swin Transformer feature extraction network consists of three main components: image blocking and linear mapping, block aggregation, and the Swin Transformer module. This structure is illustrated in Figure 2.

**Figure 3:**
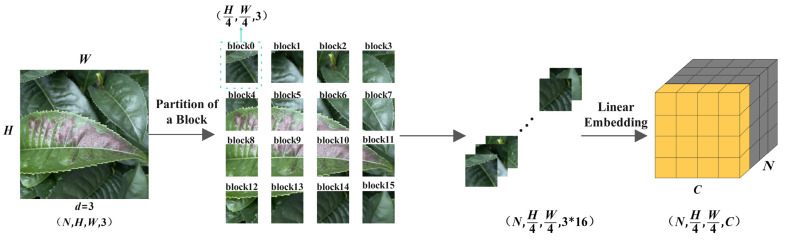
Improved structure of Patch Embedding layer.

The Patch Merging layer functions as a pooling mechanism within the backbone network, decreasing the feature map resolution and modifying the number of channels to create a hierarchical structure. This layer also helps in saving computational resources. The Patch Embedding module initially divides the image into 4 × 4 non-overlapping blocks at the beginning of the feature extraction network. Each block has a feature dimension of 4 × 4 × 3. Subsequently, a linear transformation method is used to project the feature dimension to any desired dimension, effectively converting the original two-dimensional image into a series of one-dimensional embedding vectors. These converted embedding vectors are then fed into three stages of feature extraction layers to generate hierarchical feature representations. Here, W and H represent the length and width of the input feature map,  d  denotes the channel dimension, and N  indicates the batch size. The working process of the Patch Merging layer is illustrated in Figure 3.

This study implemented two consecutive Swin Transformer modules: one based on rule partitioning windows and the other based on shift partitioning windows. The final output of the global feature extraction network was derived from the output of RSTL. The global feature modeling network leverages the strong long-distance feature dependency modeling capability of Swin Transformer to facilitate interaction between disease images and self-attention weights based on image content. This enables better extraction of color, texture, shape, and other disease image features, effectively reducing noise and artifacts. The Swin Transformer Block (STB) is an evolution of the standard multi-head self-attention in the original Transformer. One key difference lies in its implementation of local self-attention and a shift window mechanism. When processing a low-light image input of size H×W×C, the image is initially divided into local windows of size S×S and resized to $\frac{HW}{S^{2}}\times S^{2}\times C$. Subsequently, standard self-attention is computed within each window. For local window features $P\in R^{S^{2}\times C}$, the calculation formulas of Q, K, V matrices are as shown in Equation (1):

(1) $$Q=PI_{Q},K=PI_{K},V=PI_{V}$$

In the formula, $I_{Q}$, $I_{K}$, $I_{V}$ are shared projection matrices between different windows.

Generally speaking, $Q,\;K,\;V\in R^{S^{2}\times d}$, the calculation formula for obtaining the attention matrix through the self attention mechanism within the local window, is as follows:

(2) $$Attention(Q,K,V)=Softmax(QK^{T}/\sqrt{d}+B)V$$

In the formula, B represents learnable relative positional encoding.

Subsequently, a multilayer perceptron (MLP) was employed, consisting of two fully connected layers with a GELU nonlinear activation function for feature transformation. A LayerNorm (LN) layer was incorporated prior to multi-head self-attention (MSA) and MLP, with both components utilizing residual connections. The overall procedure is illustrated in Formulas (3) and (4):

(3) P=MSA(LN(P))+P

(4) P=MLP(LN(P))+P

Insufficient information exchange occurs between non-overlapping local windows. This issue can be addressed by utilizing regularly divided windows and shift divided windows alternately.

**Figure 4:**
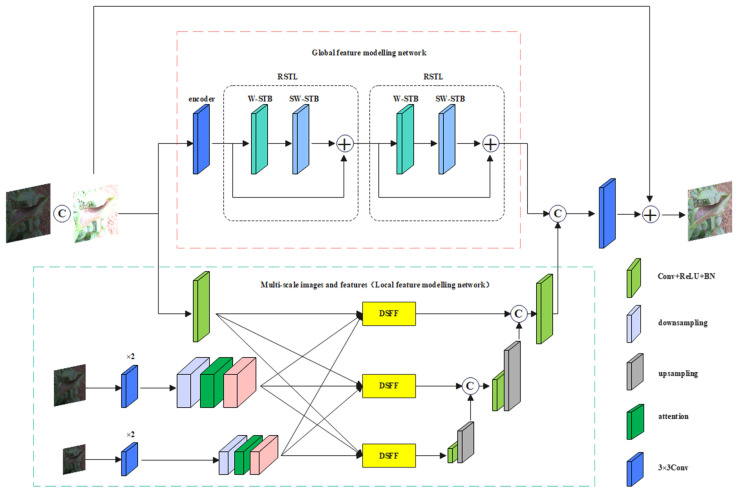
Fusion Swin Transformer multi-scale feature aggregation of attention mechanism.

**Figure 5:**
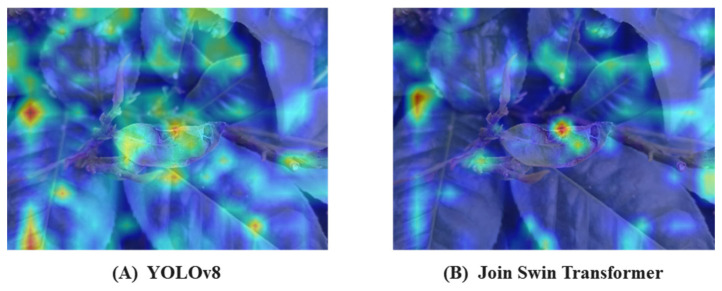
Comparison of enhancement effect of heat map before and after improvement.

Combined with the Multi-Scale Image and Feature Aggregation (MSIFA) network, the exposure of local areas in images of different scales is controlled to avoid overexposure or underexposure of the enhanced image. The construction, as depicted in the green dotted box in Figure 4, followed the MSIFA concept. The local feature modeling network within the dotted box was a U-shaped network with multiple inputs and a single output, comprising two 3 × 3 convolutional layer residual blocks and a stacked 1 × 1 convolutional layer. The residual blocks aimed to extract features from the downsampled image, while the 1 × 1 convolutional layer refined the features of the residual connection. Subsequently, the feature attention module was utilized to enhance useful feature information from the previous scale and to learn spatial and channel weights of features from the feature extraction block. To further showcase the window self-attention mechanism’s ability to capture global and local context information within the receptive field, heat map visualization was conducted using CAM-Grad. Figure 5A displays the heat map of the original YOLOv8 model, while Figure 5B shows the heat map of the model after replacing the backbone network with a Swin Transformer network.

### 2.3. Improved YOLOv8 Network Model

**Figure 6:**
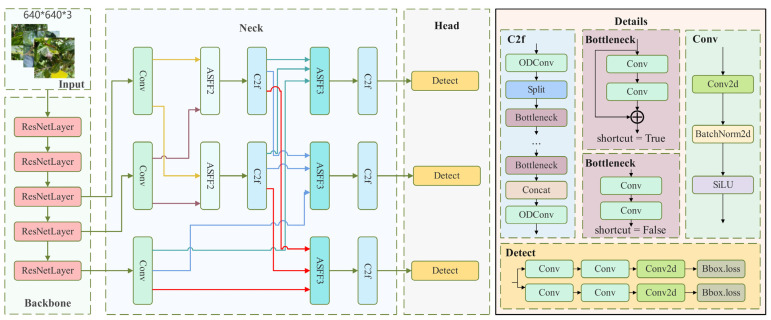
Improved YOLOv8 network architecture.

The YOLOv8 algorithm [26,27,28] is the latest version in the YOLO family, known for effectively balancing detection speed and accuracy in various scenarios, such as real-time disease detection. This algorithm comprises four main components: input end, backbone network, neck network, and prediction head. The backbone network utilizes convolution kernels, pooling layers, and activation functions to extract multi-scale and multi-level features. These features are then combined at the neck to create more informative representations. After considering factors such as model lightweight, inference speed, detection accuracy, and generalization performance, this study adopted the YOLOv8 algorithm. The improved structure of YOLOv8 is illustrated in Figure 6.

**Figure 7:**
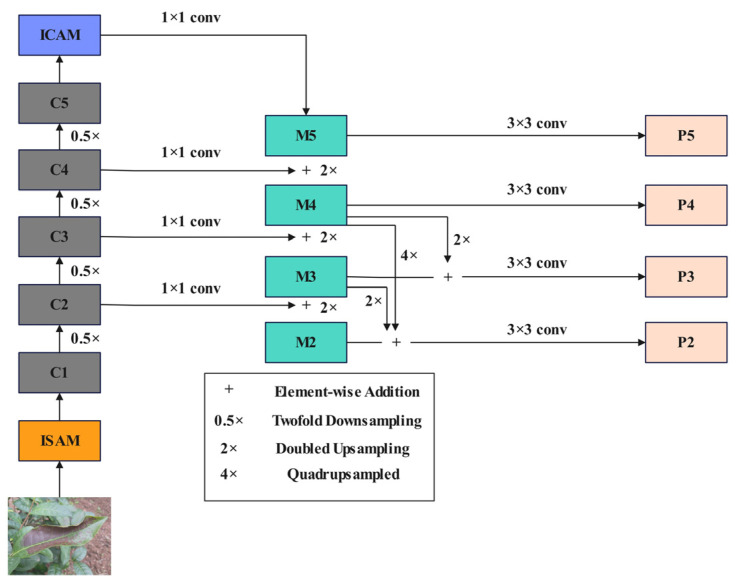
Improved ResNet50 framework diagram.

This study utilized ResNet50 as the feature extraction network in the context of YOLOv8. Enhancements to the ResNet50 and FPN structures included the integration of an improved spatial attention mechanism module (ISAM) and an improved channel attention mechanism (ICAM) within the YOLOv8 Backbone. The model architecture, depicted in Figure 7, showcases the incorporation of ISAM between the input image and the Cl feature layer as well as ICAM between C5 and M5. Additionally, ICAM and ISAM modules were integrated into the bottleneck of the C2~C5 feature layers. Within the FPN structure, feature extraction prior to fusion was denoted as {M2, M3, M4, M5}, while multi-scale features were represented as {P2, P3, P4, P5}. The upsampling method was employed for reusing M4 features in generating P3 features, and fusion of upsampled M4 features with M3 features yielded the final P3 features. Similarly, for P2 features, the bypass method was utilized to reuse M3 and M4 features, resulting in the fusion of upsampled M3, M4 features, and M2 features to produce P2 features.

#### 2.3.1. Improved Spatial Attention Mechanism

**Figure 8:**
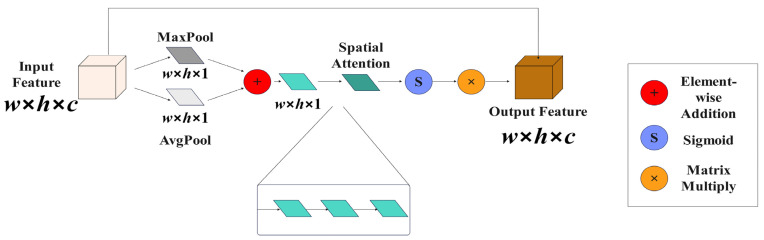
Improved spatial attention structure diagrams.

The input disease image, which has been enhanced at multiple scales and features aggregated through EnlightenGAN, contains rich and detailed information. In the ResNet50 structure, feature extraction of the input image is directly performed through maximum pooling downsampling to generate the C1 feature layer, potentially leading to loss of detailed information. To address this issue, the spatial attention (ISAM) module was enhanced. The specific structure can be seen in Figure 8. Downsampling may result in the loss of significant detailed information in the image, particularly affecting the detection of small objects. To mitigate this issue, this study employed ISAM to preprocess the image, enhancing the feature expression in key areas of the image and reducing the loss of feature information post maximum pooling.

In ISAM, the input consists of a feature layer with dimensions w×h×c. The feature layer is first compressed into w×h×1 along the channel dimension using global maximum pooling and global average pooling. The resulting compressed features are then combined through an addition operation to generate w×h×1 features. Subsequently, three 3×3 convolutions are applied to produce x×h×1 spatial attention. The spatial attention is then passed through a sigmoid function to activate it and finally multiplied with the original feature layer to obtain the w×h×c feature layer. This process can be represented by the Formula (5) as shown.

(5) $$O=S(f^{3\times 3}(f^{3\times 3}(f^{3\times 3}(M_{c}(I)+A_{c}(I)))))\times I$$

In the formula, O represents the feature layer; S represents the sigmoid activation function; $f^{3\times 3}$ represents 3 × 3 convolution; $M_{c}$ represents global maximum pooling in the channel dimension; $A_{c}$ represents global average pooling in the channel dimension; and I represents the feature layer.

#### 2.3.2. Improved Channel Attention Mechanism

**Figure 9:**
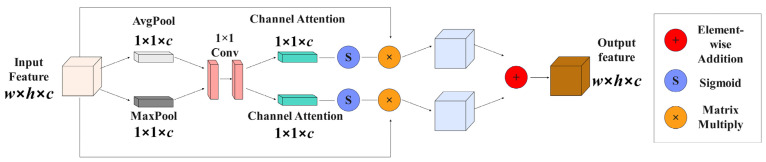
Improved channel attention structure diagram.

In the ResNet50 network, the number of channels increases significantly as the input image goes through multiple convolution and pooling operations. Prior to reducing the dimensionality of the feature layer C5, ICAM was employed to process C5 and leverage the dependency relationship between channels. This approach helped the network focus more on the semantic information of crucial channels, thereby minimizing feature loss resulting from channel reduction. Refer to Figure 9 for the visual representation of this structure.

In ICAM, the input consists of a feature layer with dimensions w×h×c. The global spatial feature information of this layer is condensed to 1×1×c using two paths: global maximum pooling and global average pooling. Subsequently, a 1 × 1 convolution operation is applied to generate global maximum channel attention and global average channel attention with dimensions 1×1×c. These attentions are then multiplied with the feature layer after activation through the sigmoid function, and the resulting features of w×h×c are obtained through addition. This process can be represented by the Formula (6):

(6) $$O=S\left(f^{1\times 1}\left(M_{s}\left(I\right)\right)\right)\times I+S\left(f^{1\times 1}\left(A_{s}\left(I\right)\right)\right)\times I$$

In the formula, O represents the feature layer; S represents the sigmoid activation function; $f^{1\times 1}$ represents 1 × 1 convolution; $M_{s}$ represents global maximum pooling in the spatial dimension; $A_{s}$ represents global average pooling in the spatial dimension; and I represents the feature layer.

**Figure 10:**
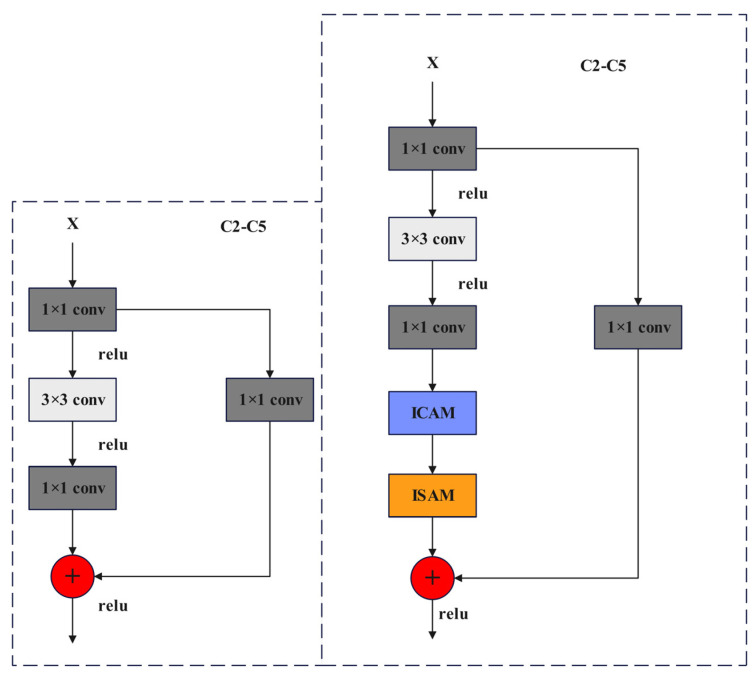
Improved feature layer before and after comparison diagram.

The enhancements to the bottleneck structure of the C2–C5 feature layer in ResNet50 are illustrated in Figure 10. The three convolution blocks on the left side of the bottleneck are denoted as the function F(x), while the one convolution block on the right side is represented as G(x), as shown in Formulas (7)–(9).

(7) $$F(x)=\&f^{1\times 1}\left(R(f^{3\times 3}(R(f^{1\times 1}(x))))\right)$$

(8) $$G(x)=\&f^{1\times 1}(x)$$

(9) $$O=\&F(x)+G\left(x\right)\;$$

In the formula, F(x) represents the output of the left branch of the bottleneck, while G(x) represents the output of the right branch. The variable $f^{1\times 1}$ denotes a 1 × 1 convolution, R represents the ReLU activation function, $f^{3\times 3}$ signifies a 3 × 3 convolution, x is the feature input, and O represents the feature output.

Incorporating ICAM and ISAM modules into the left branch of the original bottleneck can help mitigate the loss of original image details and semantic information caused by the network structure mentioned above. The improved bottleneck structure is shown in Formula (10). The feature layer improvement diagram is shown in Figure 10.

(10) $$F(x)=ISAM(ICAM(f^{1\times 1}(R(f^{3\times 3}(R(f^{1\times 1}(x))))))))$$

In the formula, F(x) is the output of the left branch of bottleneck; $f^{1\times 1}$ represents 1 × 1 convolution; R represents the Relu activation function; and $f^{3\times 3}$ represents 3 × 3 convolution.

### 2.4. Feature Fusion Network Improvement Strategy Based on ASFF

**Figure 11:**
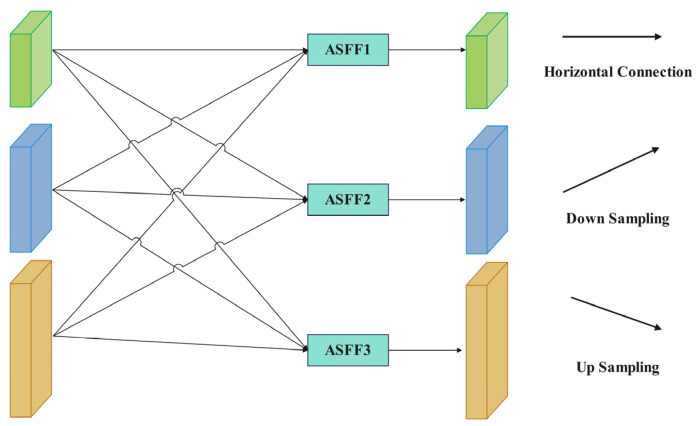
Structure diagram of adaptive spatial feature fusion network.

To address conflicts between FPN at various feature levels, this study presented the adaptive spatial feature fusion method (ASFF) [29,30], as illustrated in Figure 11. The ASFF structure effectively captures feature details across different scales and dynamically adjusts the weights of each feature layer to prioritize essential feature information.

**Figure 12:**
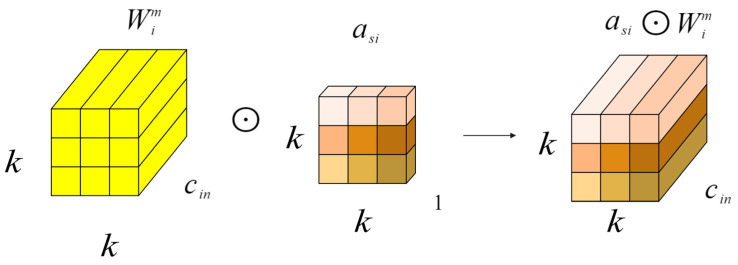
Location-wise multiplication operations along the spatial dimension.

FPN generates feature layers at multiple scales, each with varying resolutions and semantic information, denoted as Level 1, Level 2, and Level 3 in Figure 12. ASFF dynamically adjusts feature weights and spatially filters features from different levels, effectively resolving conflicts among features in FPN. The fusion process is detailed as follows:

(11) $$y_{ij}^{l}=a_{ij}^{l}\cdot x_{ij}^{1\to l}+\beta_{ij}^{l}\cdot x_{ij}^{2\to l}+\gamma_{ij}^{l}\cdot x_{ij}^{3\to l}$$

In the formula, $y_{ij}^{l}$ represents the feature vector output by the ASFF network. The input feature vectors $x_{ij}^{1\to l}$, $x_{ij}^{2\to l}$, $x_{ij}^{3\to l}$ correspond to the three feature maps at different levels up to the l-th layer. The parameters $a_{ij}^{l}$, $\beta_{ij}^{l}$, and $\gamma_{ij}^{l}$ are learnable parameters for the three levels of feature maps. These feature maps with weight parameters from Level 1, Level 2, and Level 3 are obtained through 1 × 1 convolutions, where the sum of the weight parameters a, β, and γ is 1. After normalization, the weight parameter values range from 0 to 1.

### 2.5. Neck Network with ODConv

**Figure 13:**
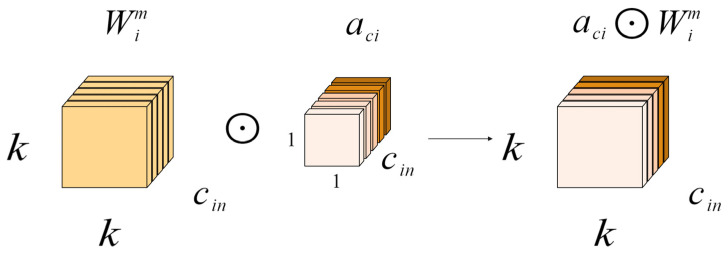
Channel-wise multiplication operations along the input channel dimension.

**Figure 14:**
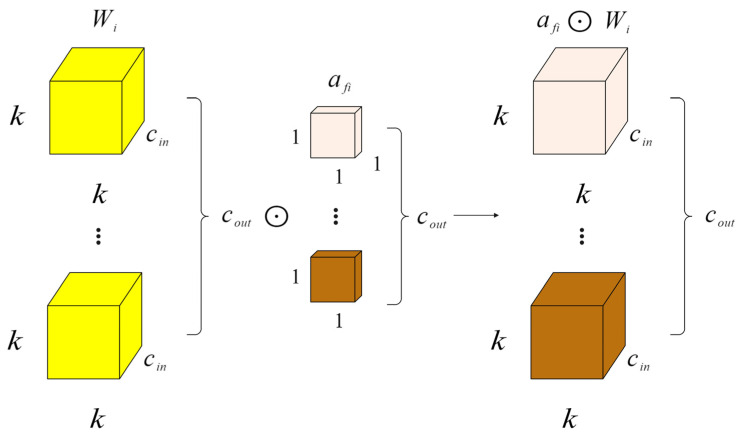
Filter-wise multiplication operations along the output channel dimension.

**Figure 15:**
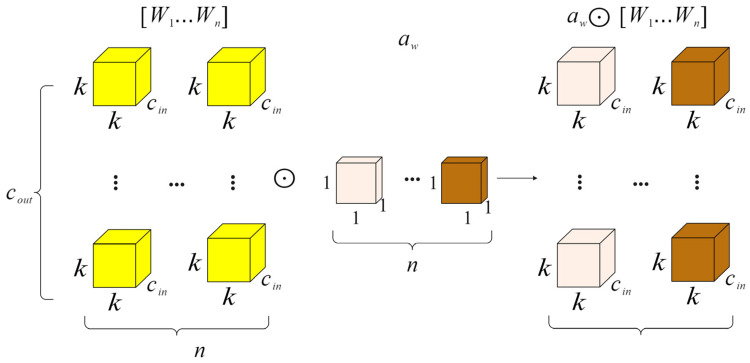
Kernel-wise multiplication operations along the kernel dimension of the convolutional kernel space.

In order to enhance the speed and performance of neural networks, we proposed a new dynamic convolution design called full-dimensional dynamic convolution (ODConv) [31,32,33]. ODConv can easily be integrated into the existing YOLOv8 network, improving the feature extraction capabilities of deep convolutional neural networks. Serving as an extension of CondConv, ODConv builds upon CondConv by incorporating all four dimensions of kernel space—including air space, input channel, and output channel—in a parallel manner. By introducing four types of attention to the accumulation kernel and gradually applying these attentions to the respective convolution kernels, ODConv significantly boosts the ability to extract disease features at each convolution layer. The structural illustration of gradually multiplying the four types of attention in ODConv to the convolution kernel can be seen in Figure 12, Figure 13, Figure 14 and Figure 15.

Mathematically, the convolution kernel can be defined for the dynamic convolution operation at a specific spatial location, considering different input channels, different output channels, and the overall convolution kernel, as shown in Equation (12).

(12) $$y=\left(\alpha_{w1}W_{1}+\ldots +\alpha_{wn}W_{n}\right)*x$$

In the formula, $x\in R^{h\times w\times c_{in}}$ and $y\in R^{h\times w\times c_{out}}$ represent the input features and output features, respectively, where $c_{in}$/$c_{out}$ channels have a height of h and width of w. $W_{i}$ represents the i-th convolution kernel composed of $c_{out}$ filters, with $W_{i}^{m}\in R^{k\times k\times c_{in}}$; $\alpha_{wi}\in R$ is the attention scalar. ODConv can be defined by Formula (13).

(13) $$y=(\alpha_{w1}⊙\alpha_{f1}⊙\alpha_{c1}⊙\alpha_{s1}⊙W_{1}+\ldots +\alpha_{wn}⊙\alpha_{fn}⊙\alpha_{cn}⊙\alpha_{sn}⊙W_{n})*x$$

The attention scalar of the convolution kernel $W_{i}$ is denoted as $\alpha_{wi}\in R$, similar to Formula (8). Additionally, $\alpha_{si}\in R^{k\times k},\alpha_{ci}\in R^{c_{in}}$, and $\alpha_{fi}\in R^{c_{out}}$ represent the newly introduced attention points along the spatial, input channel, and output channel dimensions of the convolution kernel $W_{i}$. The symbol ⊙ signifies the multiplication operation across different dimensions of the kernel space. The values of $\alpha_{si}$, $\alpha_{ci}$, $\alpha_{fi}$, and $\alpha_{wi}$ are computed by the multi-head attention module $\pi_{i}(x)$.

In principle, these four types of attention are complementary. By progressively applying various forms of attention across different dimensions, such as position, channel, filter, and kernel, the convolution operation can capture diverse contextual information, leading to improved performance. ODConv, utilizing fewer convolution kernels, is able to achieve comparable or superior results compared to CondConv and DyConv.

### 2.6. Loss Function Optimization

The regression loss function of the bounding box is a critical aspect in object detection. In the initial iterations of the YOLO series, the Generalized IoU Loss was employed as the loss function [34,35]. The calculation formula for GIoU is represented by Formula (14).

(14) $$GIoU=1-IoU+\frac{\left|S-A\cup B\right|}{\left|S\right|}$$

where IoU refers to the intersection and concurrency ratio of the true frame to the predicted frame.

In the traditional IoU loss function, when the predicted box and the real box do not intersect, the IoU value is always 1, and the loss function output is always 0. GIoU addresses this issue by introducing the minimum convex closed box area S of the predicted box A and the real box B, ensuring that the loss can still decrease even when A and B do not intersect. However, challenges remain, such as the inability to measure the positional relationship between two boxes when they are contained within each other as well as the computational complexity and slow convergence when the prediction box is aligned horizontally or vertically. The latest YOLOv8 model introduces CIoU as the primary loss function, replacing GIoU optimization with a direct minimization of the distance between the two target frames. This approach resolves issues of large losses and slow convergence in GIoU when the frames are distant and enhances detection accuracy for overlapping dense targets by adjusting aspect ratio parameters. The CIoU calculation formula is presented in Equation (15).

(15) $$CIoU=1-IoU+\frac{\rho^{2}(A,B)}{c^{2}}+\alpha \nu$$

The formula $\rho^{2}(A,B)$ represents the Euclidean distance between the center points of two frames. Here, c  denotes the diagonal length of the frames, and αν signifies the influence factor of the aspect ratio of the frames. The parameters α and ν are further divided into balance proportion coefficients and considerations for the consistency of the proportions of the frames.

(16) $$\alpha =\frac{\nu }{(1-\mathrm{IoU})+\nu }$$

(17) $$\nu =\frac{4}{\pi^{2}}(arctan\frac{w^{gt}}{h^{gt}}-arctan\frac{w}{h}{)}^{2}$$

In the formula, w, $w^{gt}$, h, $h^{gt}$ are the width and length of the two frames, respectively. Tea leaf diseases are dense, small objects in images, and the detection performance can be easily reduced by the position deviation of small objects when using the intersection-over-union ratio (IOU) expansion index.

Tea disease severity detection is a single-category detection task, focusing more on classification and accurate positioning during the detection stage. Due to the presence of overlapping and multiple disease targets in practical detection scenarios, EIoU is introduced as a replacement for CIoU. Building upon CIoU, EIoU further emphasizes the actual difference in width and height, weighing its confidence to minimize the disparity between the real and predicted frames. This approach accelerates model convergence. The EIoU loss function comprises three components: overlap loss calculation, center point distance loss calculation, and width and height loss calculation. After enhancing Formula (14), it is presented in (18):

(18) $$EIoU=1-IoU+\frac{\rho^{2}(b,b^{gt})}{L^{2}}\&+\frac{\rho^{2}(w,w^{gt})}{L_{w}^{2}}+\frac{\rho^{2}(h,h^{gt})}{L_{h}^{2}}$$

**Figure 16:**
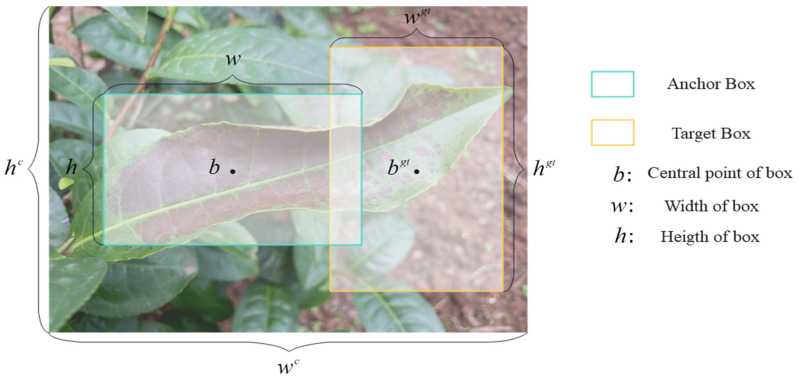
Graphical representation of EIoU calculation factors.

In the formula, b and $b^{gt}$ represent the center points of the two frames; L is the diagonal distance of the minimum circumscribed rectangle of the two frames; and $L_{w}$, $L_{h}$ are the width and length of the circumscribed rectangle of the two frames, respectively. The expression $\frac{\rho^{2}(b,b^{gt})}{L^{2}}$ reflects the center point distance between the regression frame, and the real frame $\frac{\rho^{2}(w,w^{gt})}{L_{w}^{2}}$ and $\frac{\rho^{2}(h,h^{gt})}{L_{h}^{2}}$ reflects the difference in width and height between the regression box and the real box, as shown in Figure 16.

### 2.7. Tea Disease Image Acquisition

**Figure 17:**
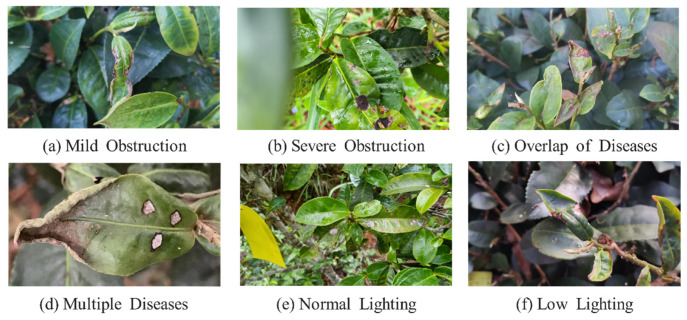
Examples of tea disease samples.

The disease dataset was collected at Hekai Base, Menghai County, Xishuangbanna Prefecture, Yunnan Province, China (21.5 N, 100.28 E), using a Canon EOS 90D camera. The dataset consisted of images of Yunnan’s unique large-leaf sun-dried green tea. Large-leaf tea in Yunnan shows a seasonal incidence pattern due to the region’s moderate temperature and high humidity, with autumn being the most common season for disease occurrence in tea gardens. The large-leaf sun-dried green tea in Yunnan represents over 80% of the domestic tea planting area. A total of 4300 images were initially collected, of which 2700 images were selected after filtering out photos with poor quality. The dataset comprised 3743 labeled images of three diseases: tea leaf blight, tea white spot disease, and tea coal disease. It covered scenarios with overlapping occlusion and coexistence of multiple diseases under low-light conditions. The dataset included images with varying levels of occlusion, disease overlap, and different light intensities to enhance the diversity of large-leaf tea disease detection in complex environments. For example, Figure 17 illustrates tea disease samples. The dataset was divided into 80% for training and 20% for validation purposes.

**Figure 18:**
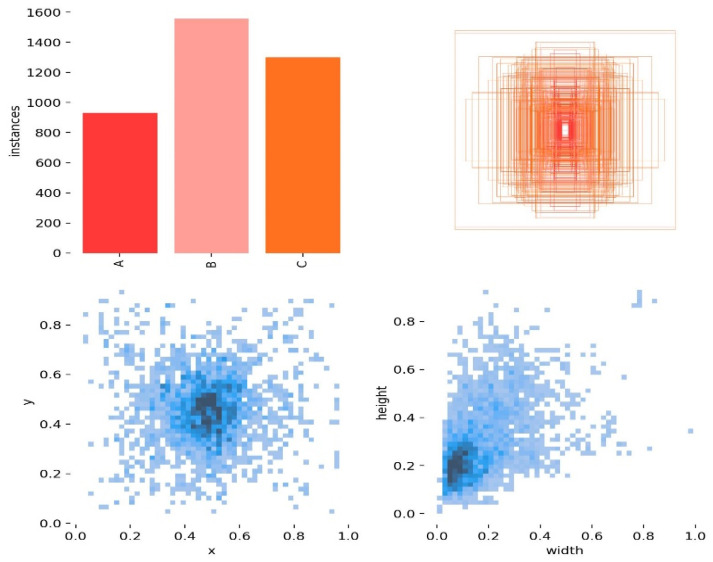
Number and size distribution of each tea disease category.

The training set was annotated using the image data annotation software LabelImg, with a focus on tea disease targets. Annotations were made based on the smallest rectangle surrounding the disease, with the aim of minimizing background inclusion. The saved comments were in XML format. Figure 18 displays the visual analysis of the tea disease annotation file, revealing varying sizes of target boxes with ratios mostly falling between 0.06 and 0.3. The top two figures in Figure 18 represent the histograms of tea leaf blight, tea white spot disease, and tea coal disease and the length and width of each label box, while the following two figures represent the distribution of diseases in the image in proportion to the width and height of labels. The presence of numerous small disease targets poses a challenge for detection.

## 3. Results and Discussion

### 3.1. Experimental Platform and Parameter Configuration

For model training, this study utilized an Intel(R) Core(TM) i7-11700 processor and an RTX3090 graphics card with 16 GB of memory. The software environment consisted of CUDA version 11.8, Python 3.8, and Pytorch version 2.0.0. Details of the computer software and hardware training environment can be found in Table 1 (Intel Corporation, Santa Clara, CA, USA; NVIDIA Corporation, Santa Clara, CA, USA).

In order to ensure the effectiveness of the comparative experiment, standardized parameters were utilized during the training phase. The study opted for an image size of 640 × 640 for training, employed a gradient-based SGD optimizer for model optimization, and initialized the learning rate at 0.01. Moreover, to enhance the stability and convergence speed during model training, default values were set for the optimizer momentum (0.937) and weight decay coefficient (0.0005), with 1000 iterations and a batch size of 16. These hyperparameters were selected based on prior experimental findings to ensure optimal model performance across various conditions. Refer to Table 2 for details.

### 3.2. Tea Disease Severity Rating

The disease index is utilized to assess the severity of tea diseases. Following the onset of symptoms, a five-point survey method is employed to categorize the severity of leaf diseases into three levels. In the experiment, tea leaf blight, tea white spot disease, and tea soot disease were classified as mild, moderate, and severe disease grades. Specifically, mild, moderate, and severe tea leaf blight were denoted as A, B, and C, respectively. Similarly, tea white spot disease was categorized as D, E, and F for mild, moderate, and severe cases, while tea sooty disease was labeled as G, H, and I for mild, moderate, and severe symptoms, resulting in a total of 9 categories. The formula is depicted in Equation (19).

(19) $$DI(\%)=\frac{\sum (x\times f)}{n\times \sum f}\times 100$$

In the formula, x represents the level value of each gradient, f represents the number of blades of each gradient, and the highest gradient value of n is 3.

### 3.3. Indicators for Model Evaluation

When analyzing the experimental results, this study employs accuracy (precision), recall (recall), F1 balance score, average precision (AP), mean average precision (mAP), and frames per second (FPS) as performance evaluation metrics for the model. The intersection ratio threshold is set at 0.5, with prediction boxes below the threshold considered incorrect predictions, as demonstrated in Equations (20)–(25) [31,32].

(20) $$Precision=\frac{T_{P}}{T_{P}+F_{P}}$$

(21) $$Recall=\frac{T_{P}}{T_{P}+F_{N}}$$

(22) $$F1=2\times \frac{Precision\times Recall}{Precision+Recall}$$

(23) $$AP=\int_{0}^{1}Precision\left(Recall\right)dRecall$$

(24) $$mAP=\frac{\sum_{i=1}^{C}AP(i)}{C}$$

(25) $$FPS=\frac{1000}{time}$$

The formula is defined as follows: $T_{P}$ represents the number of images in the test set where the tea disease image category is correctly recognized by the model, $F_{P}$ represents the number of images where tea disease images of other categories are incorrectly recognized as the current category, and $F_{N}$ represents the number of images where the current category of tea disease images is incorrectly recognized as other categories. C is the number of categories of tea diseases in the test set. FPS represents the number of images processed by the model per second, and time refers to the duration required by the model to process a single image, calculated in milliseconds.

### 3.4. Experimental Results Obtained from a Self-Built Dataset Using an Improved Version of YOLOv8

**Figure 19:**
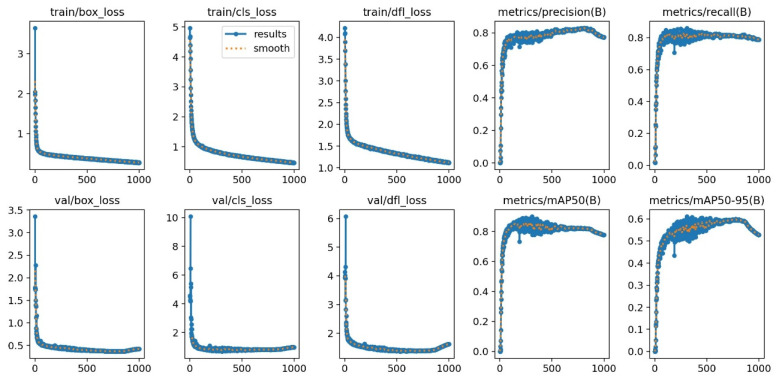
Performance values for the YOLOv8-ASFF model.

In this study, the model training was conducted for 1000 rounds with an automatic stopping mechanism implemented when the average accuracy plateaued. The training process concluded after approximately 980 rounds, at which point YOLOv8-ASFF provided the training results on the custom dataset. The performance metrics of the training and validation sets are depicted in Figure 19.

The study presents an analysis of the box loss, object loss, and classification loss of the enhanced YOLOv8-ASFF model. The graphs in the initial three columns depict the progression of loss over time during training, with the *X*-axis indicating training duration and the *Y*-axis showing the loss value. The graphs show a consistent decrease in loss value as training advances, eventually stabilizing. Notably, there is no evidence of overfitting during the network training process. The results indicate that the YOLOv8-ASFF model demonstrates strong fitting performance and stability. The final two columns display the PR curve, with the *X*-axis representing training time and the *Y*-axis showing precision and recall. These curves evaluate object detection performance based on changes in the confidence threshold. A curve value closer to 1 signifies higher model confidence. The analysis in Figure 19 demonstrates the effectiveness of the YOLOv8-ASFF model.

### 3.5. Dataset Training of YOLOv8

In order to evaluate the impact of YOLOv8-ASFF on detecting tea leaf blight, tea white spot disease, and tea sooty disease in Yunnan large-leaf tea, four sets of comparative experiments were conducted. The experiments compared YOLOv8-ASFF with four established mainstream network models, including YOLOv8 [35], YOLOv5 [36], CornerNet [37], and SSD [38]. To ensure the reliability of the model test results, the hardware equipment and software environment were kept consistent throughout the study. The detection performance parameters of the four networks are presented in Table 3.

Compared with the Information Entropy Masked Vision Transformer model studied by Jiahong Zhang [39], the accuracy of tea disease detection is 1.48 percentage points higher. Compared with the genetic optimization neural network studied by Zhang Shuaitang [40], the accuracy of tea disease detection was 1.09 percentage points higher.

**Figure 20:**
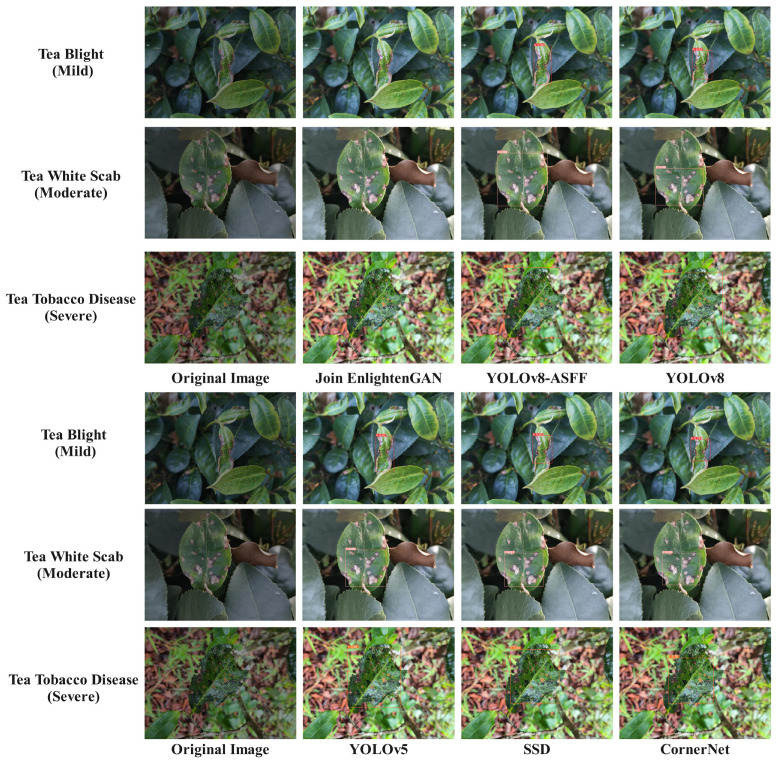
Comparison of recognition effects of different networks.

The three types of tea diseases images included mild tea leaf blight, moderate tea white spot disease, and severe tea sooty disease. *Alternaria alternata*, *Phyllosticta theaefolia Hara*, and *Neocapnodium theae Hara* were the main scientific pathogens of tea blight, tea white star disease, and tea sooty disease and were chosen for detection tests, as depicted in Figure 20. The research revealed that the YOLOv8-ASFF-based network achieved superior recognition accuracy and a lower miss detection rate.

### 3.6. Visual Recognition of Heat Map

In order to elucidate the process of tea disease severity detection using the YOLOv8-ASFF network model, this study employs the visualization technique known as gradient weighted class activation mapping (Grad-CAM). The study compares the recognition performance of the YOLOv8-ASFF network model across different levels of three tea diseases. In the Grad-CAM visualization method, the fusion weight of the target feature map is depicted as a gradient, and the global average of the gradient is utilized to calculate the weight. Subsequently, after obtaining the weights of all feature maps for each disease category, these weights are combined to generate a heat map.

**Figure 21:**
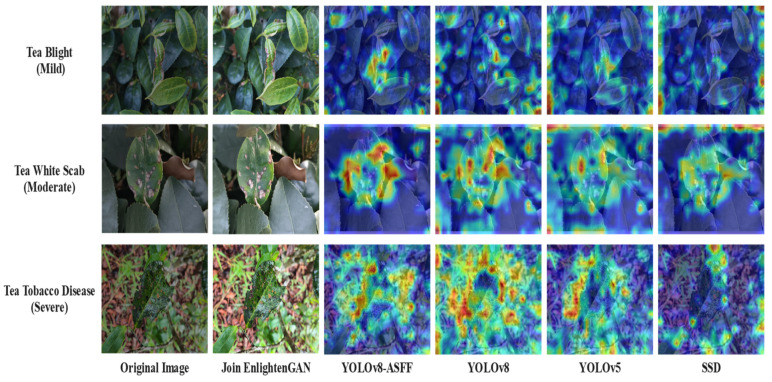
Disease severity test result graph.

Heat maps visually depict the model’s focus during feature extraction, with warmer colors indicating higher attention. In Figure 21, Grad-CAM illustrates the progression of three diseases from mild to severe. The YOLOv8-ASFF network model accurately focuses on images of various disease types, with the thermal area mainly concentrated on key features of leaf diseases and some irrelevant features, unaffected by background factors. This further confirms the efficacy of the proposed network in detecting the severity of tea diseases.

### 3.7. Ablation Experiment

In order to investigate the performance enhancement of the YOLOv8 model achieved by integrating the ResNet50 network, adaptive spatial feature fusion module (ASFF), and ODConv module as well as to validate the efficacy of each component, ablation experiments were conducted. The analysis and research focused on the training process of YOLOv8-R, YOLOv8-A, YOLOv8-O, YOLOv8-RA, YOLOv8-RO, YOLOv8-AO, and YOLOv8-RAO models in terms of mAP@0.5 and mAP@0.95 experimental data, parameters, FLOP, and FPS.

After utilizing the ResNet50 model to enhance the backbone network of the YOLOv8 model, an analysis of test results in Table 4 reveals a significant increase in the number of model parameters. However, both mAP@0.5 and mAP@0.5:0.95 show improvement. Furthermore, upon integration into the ODConv module, there is a respective increase of 0.69% and 0.61% in the number of model parameters. Despite increases in model parameters resulting from improvements to the backbone network model and the addition of the adaptive spatial attention mechanism and ODConv module, there is a reduction in floating point calculations while effectively increasing accuracy with mAP@0.5 and mAP@0.5-0.95, showing improvements by 3.72 and 1.85 percentage points, respectively. Additionally, the final detection speed of the model reaches 117 FPS, meeting real-time requirements.

## 4. Conclusions

Based on the YOLOv8 model, an improved tea disease severity detection model named EnlightenGAN-YOLOv8-ASFF was proposed in this paper. The proposed model aims to achieve the rapid, accurate, and non-destructive detection of disease severity under low-light-intensity conditions. The study provides valuable theoretical insights for the advancement of smart tea garden management. Addressing challenges posed by extreme tea garden environments, such as rainfall, darkness, and light intensity, remains a key research focus. The article enhances the EnlightenGAN network to generate high-quality disease images under low-light conditions, expands tea disease data, improves spot characteristics and detailed textures in low-light settings, and offers valuable methods for subsequent disease detection.

To address the issue of small feature differences in disease severity levels and challenges in classifying fine-grained disease images, this study utilizes ResNet50 as the backbone network for the YOLOv8 model. Channel and spatial attention modules are incorporated at various levels of the ResNet50 structure to leverage distinct features. Specifically, the neck layer is designed to extract crucial details from similar disease feature maps, with the addition of an adaptive weighted feature fusion module (ASFF) and the replacement of Conv convolution with full-dimensional dynamic convolution (ODConv). This enhancement allows for better differentiation across dimensions and, when combined with the EIoU loss function, results in improved detection and localization accuracy. The YOLOv8-ASFF model achieves a precision rate of 87.47%, recall rate of 89.17%, F1 value of 88.31%, and 95.8% accuracy in estimating disease severity for tea blight, tea white spot disease, and tea sooty disease. A comparative analysis with other detection models, such as CornerNet, SSD, YOLOv5, and YOLOv8, demonstrates superior target-recognition performance while maintaining recognition speed. YOLOv8-ASFF exhibits an average accuracy increase of 16.22%, 10.87%, and 6.07% over the aforementioned models, with a recognition speed of 89 frames/second and enhanced recognition accuracy. All evaluation indicators have improved, indicating that this model significantly enhances the YOLOv8 network’s ability to detect disease areas in images. It outperforms CornerNet, SSD, YOLOv5, and YOLOv8 models in terms of accuracy, with lower rates of missed detections and false alarms.

The improved YOLOv8-ASFF method proposed in this study has an efficient and accurate detection effect on tea blight, tea white spot, and tea smoke spot with different disease degrees. Tea diseases can be identified by analyzing the shape, size, and distribution of the lesions. The combination of heat map visualization in this approach not only helps to identify the onset of the disease early but also to view the severity of the disease and implement appropriate prevention and control measures in a timely manner. It completes the intelligent management of tea garden diseases.

## Figures and Tables

**Table 1:** Computer hardware and software training environment.

Configuration	Configuration Name	Detailed Information
Hardware configuration	CPU	Intel(R)CORE(TM)i7-11700
	RAM	16 GB
	GPU	NVIDIA RTX 3090
	Graphics memory size	12 GB
Software configuration	Operating system	Windows 11
	Python version	3.8
	Deep learning frameworks	Pytorch 2.0.0
	CUDA	11.8

**Table 2:** Hyperparameters for training.

Hyperparameters	Value
Gradient optimiser	SGD
Initial learning rate	0.01
Number of training batches	16
Optimiser momentum	0.937
Optimiser weight decay factor	0.0005
Image size	640 × 640
Number of iterations	1000

**Table 3:** Identification effect parameters of different models.

Target Detection Networks	mAP(%)	Precision(%)	Recall(%)	F1 Score(%)	Detection Speed/FPS
CornerNet	79.04	78.36	80.18	79.26	143
SSD	84.39	77.92	81.70	79.77	161
YOLOv5	89.19	80.39	85.66	82.94	119
YOLOv8	91.54	85.00	85.40	85.20	84
YOLOv8-ASFF	95.26	87.47	89.17	88.31	89

**Table 4:** Ablation experiment of YOLOv8 model based on self-built dataset.

Structural Model	mAP@0.5/%	mAP@0.5-0.95/%	Parameters/	mAP@0.5/%	mAP@0.5-0.95/%
YOLOv8	91.54	67.42	3.1572	8.9	69.3
YOLOv8-R	93.62	68.21	26.0785	74.3	75.19
YOLOv8-A	93.57	68.03	3.1441	9.4	84.75
YOLOv8-O	92.23	67.56	3.1576	8.9	79.49
YOLOv8-RA	94.77	68.94	25.6806	74.2	85.64
YOLOv8-RO	94.32	68.73	26.0785	74.3	93.73
YOLOv8-AO	93.93	68.45	3.1442	9.4	105.29
YOLOv8-RAO	95.26	69.27	25.6806	74.2	117

## Data Availability

The original contributions presented in the study are included in the article. Further inquiries can be directed to the corresponding authors.
